# Minimal invasive extracorporeal circulation: A bibliometric network analysis of the global scientific output

**DOI:** 10.1177/02676591241269729

**Published:** 2024-09-17

**Authors:** Jacopo D’Andria Ursoleo, Rosario Losiggio, Margherita Licheri, Gaia Barucco, Stefano Lazzari, Carolina Faustini, Fabrizio Monaco, Collaborators

**Affiliations:** Department of Anesthesia and Intensive Care, 9372 IRCCS San Raffaele Scientific Institute, Milan, Italy

**Keywords:** bibliometric network analysis, cardiac surgery, cardiopulmonary bypass, minimal invasive extracorporeal circulation, mini cardiopulmonary bypass

## Abstract

**Introduction:**

Minimal Invasive Extracorporeal Circulation (MiECC) has recently emerged as a more ‘physiologic’ alternative to conventional extracorporeal circulation. However, its adoption is still limited due to lack of robust scientific evidence and ongoing debate about its potential benefits. This bibliometric analysis aims to analyze the scientific articles on MiECC and identify current research domains and existing gaps to be addressed in future studies.

**Methods:**

Pertinent articles were retrieved from the Web of Science (WOS) database. The search string included ‘minimal invasive extracorporeal circulation’ and its synonyms. The VOSviewer (version 1.6.17) software was used to conduct comprehensive analyses. Semantic and research networks, bibliographic coupling and journal analysis were performed.

**Results:**

Of the 1777 articles identified in WOS, 292 were retrieved. The trend in publications increased from 1991 to date. Most articles focused on transfusion requirements, acute kidney injury, inflammatory markers and cytokines, inflammation and delirium, though the impact of intraoperative optimal fluid and hemodynamic management as far as the occurrence of postoperative complications were poorly addressed. The semantic network analysis found inter-connections between the terms “cardiopulmonary bypass”, “inflammatory response”, and “cardiac surgery”. *Perfusion* contributed the highest number of published documents. The most extensive research partnerships were between Germany, Greece, Italy, and England.

**Conclusions:**

Notwithstanding the scientific community’s growing interest in MiECC, crucial topics (i.e., the best anesthetic management and intraoperative need for inotropes, vasopressors and fluids) still require more comprehensive exploration. This investigation may prove to be a useful tool for clinicians, scientists, and students concerning global publication output and for the use of MiECC in cardiac surgery.

## Graphical abstract

**Graphical Abstract:**
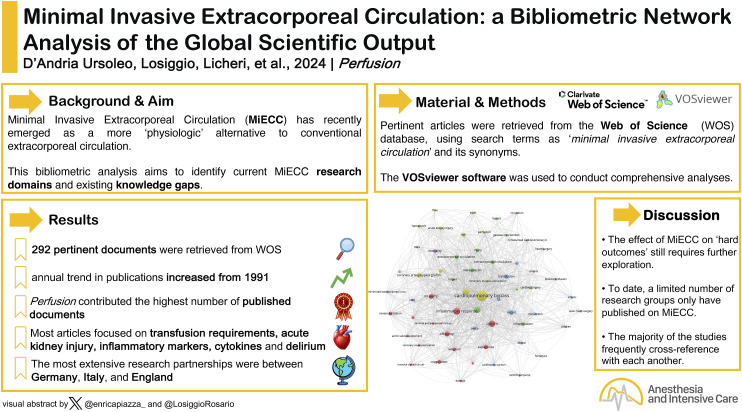

## Introduction

Cardiopulmonary bypass (CPB) poses a significant risk of complications and adverse events, contributing to morbidity and mortality in patients undergoing cardiac surgery.^1,2^ For nearly five decades, CPB technology remained largely unchanged.^3^ However, in the early 21st century, in line with the increasingly popular trend towards minimally invasive surgical approaches in cardiac surgery, a corresponding shift in extracorporeal circulation (ECC) techniques took place, with the introduction of minimal invasive extracorporeal circulation (MiECC). Nevertheless, the adoption of such technology in everyday clinical practice is still limited, with only 10%–20% of cardiothoracic surgical centers in the UK using it.^4,5^

The lack of a widespread adoption of this perfusion technique may be primarily attributed to its time- and resource-consuming nature, which may discourage practitioners from using it in the absence of robust scientific evidence. Additionally, scientific debate on the potential reduction in blood loss, blood activation, myocardial infarction, postoperative arrhythmias, cerebrovascular events, and mortality following MiECC use is still ongoing.^6^ Consequently, several unaddressed research gaps persist within the context of MiECC. Furthermore, before a technique can become a widely accepted as standard clinical practice, even if it offers theoretical superior benefits, it must be consistently reproducible. As such, evidence shows that clinicians still limit the use of MiECC in cardiac surgery.^4,5^

While meta-analyses provide robust evidence by summarizing results from multiple studies, randomized controlled trials (RCTs) show cause and effect relationships, and systematic reviews give a comprehensive overview of all available evidence, bibliometrics involve employing statistical methods to assess large quantities of bibliometric data from global scientific outputs from the perspective of their intellectual structure.^7^ Aided by the results of the analysis of many studies on a specific subject, researchers can efficiently survey a vast body of literature, summarizing insights from thousands of articles in a short time. Given the crucial role undeniably held by research in laying the foundation for treatment decisions and considering the escalating publication volume, a rise in the number of studies based on statistical and bibliometric analysis across various important medical issues was recently observed.^8–10^ To date, a bibliometric study of the growing body of published literature on MiECC is yet to be performed.

Therefore, there are two primary objectives of this study. First, to delineate the major clinical domains where MiECC is employed, providing through a bibliometric network analysis an overview of existing published research and emerging trends in the field while identifying the most influential researches, authors, journals, institutions and countries. Secondly, to unveil potential research gaps and cross-country collaborations, ultimately seeking to provide insights to be implemented in future studies, promote more collaborative networks, and stimulate further translational research efforts.

## Material and methods

### Data collection

The research was conducted following the methodology described in previous bibliometric studies.^11,12^ In order to perform a comprehensive literature review on MiECC, we used the Web of Science (WOS) online database (by Clarivate Analytics) and retrieved pertinent articles using a search string that included terms such as ‘miniaturized extracorporeal circulation’, ‘mini-extracorporeal circulation’, ‘minimized extracorporeal circulation’, ‘mini-cardiopulmonary bypass’, ‘minimally invasive cardiopulmonary bypass’, ‘miniaturized cardiopulmonary bypass’, ‘minimized perfusion circuit’, ‘minimized extracorporeal life support system’ or ‘minimized CPB’. The Boolean operator ‘OR’ was used to combine the different search terms and the Topic (Title, abstract and keywords) only part of the studies was searched for publications. Neither language nor year of publication limitations were imposed. All data were accessed on November 30, 2023, and exported as a Microsoft Excel (.xlsx) worksheet.

### Research methods

Two experienced investigators (R.L. and J.D.U.) independently screened the search results at title/abstract level for eligibility. Following careful assessment of complete articles, the final selection of studies to be retrieved was made. Disagreement was resolved by means of discussion under the supervision of a third, senior investigator (F.M.).

Pertinent information fulfilling research requirements was extracted from the retrieved documents, including: year of publication, journal name and metrics (impact factor and quartile), title, authors, topics (according to WOS categories), keywords and total number of citations. The source for the journal metrics was Journal Citation Reports^TM^ 2022 (by Clarivate Analytics).

The trend in the annual number of published documents was calculated manually on the Microsoft Excel exported worksheet.

The VOSviewer (version 1.6.17) software tool was employed to analyze several features of the retrieved articles as below, conduct a literature analysis and comprehensive knowledge bibliometric network visualization on the topic. The following features were examined:

• Co-occurrence of keywords, which involved analyzing potential relationships (interconnection) between terms using a co-occurrence or semantic network.^13^
• Co-citation, also referred to as “bibliographic coupling”. It measured the frequency with which two documents were cited together by other documents. This type of analysis was made using both ‘source’ (journal) and ‘author’ as a unit of analysis.^14^
• Co-authorship analysis (by countries), to evaluate the collaborative publishing efforts among institutions and countries.

Additionally, a supplementary analysis to dissect the most productive organizations and their collaborations was conducted.

## Results

**Figure 1.**
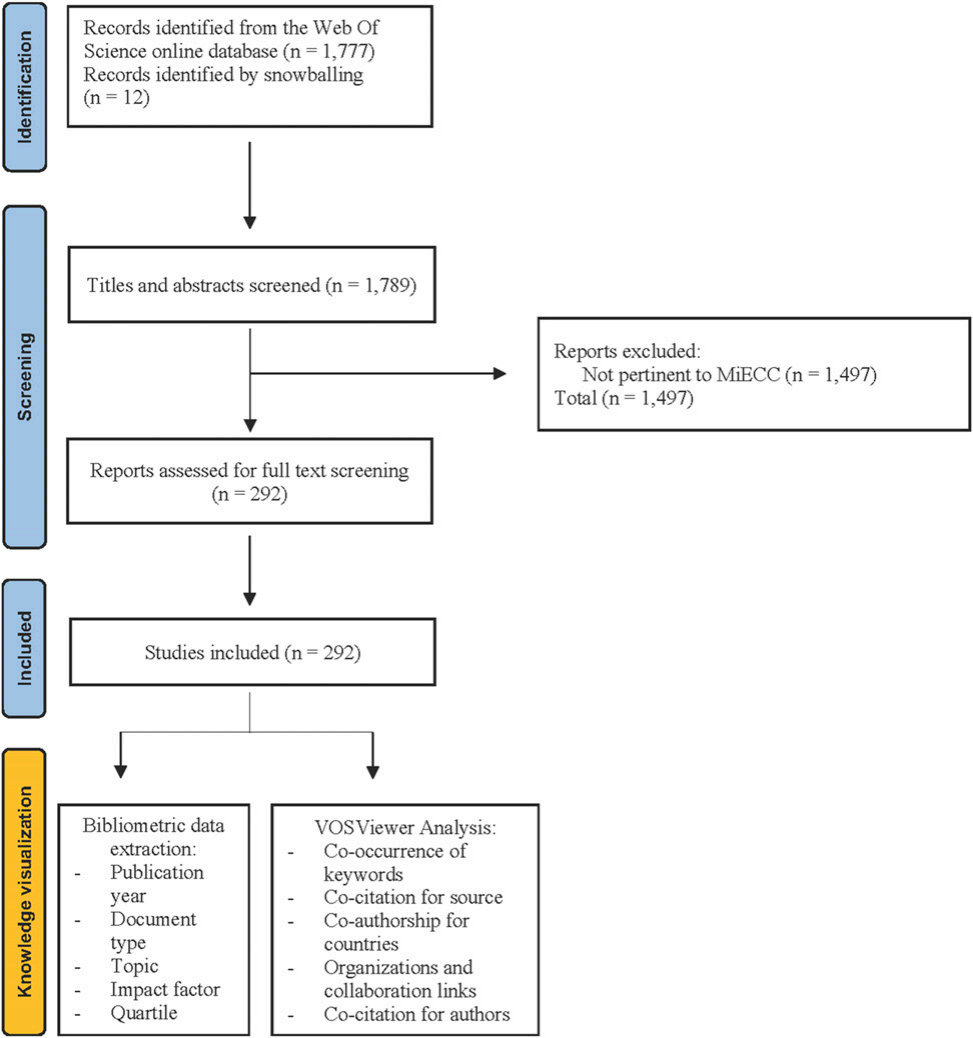
Flowchart of the study selection process. MiECC: minimal invasive extracorporeal circulation.

Our search strategy conducted on the online WOS database yielded a total of 1777 documents (from inception until November 30, 2023). The process of studies identification, screening and inclusion is summarized in Figure 1. A total of 1497 documents were not relevant to the research topic and excluded. Furthermore, 12 additional documents were added by means of snowballing from reviews and consensus documents pertinent to the research topic. Consequently, a total of 292 documents were retrieved and included in the present bibliometric network analysis.

**Figure 2.**
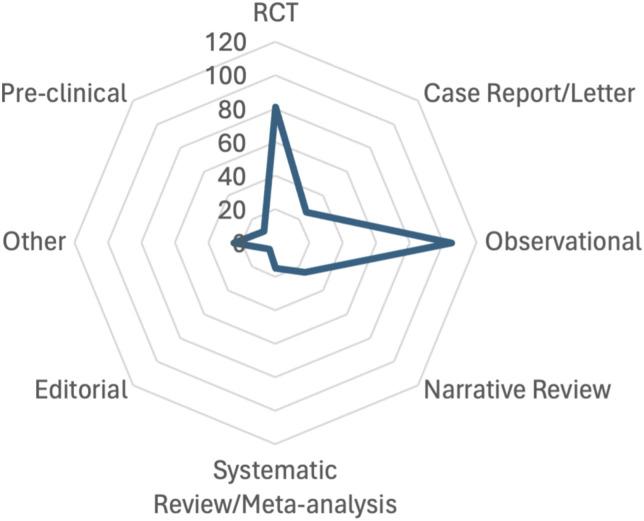
Types of the study. RCT: randomized controlled trial.

In terms of study types, within the entire dataset analyzed (*n* = 292), the most prevalent categories were observational studies (*n* = 105), randomized controlled trials (*n* = 81), and narrative reviews (*n* = 25) (Figure 2).

### Bibliometric analysis of publication output

**Figure 3.**
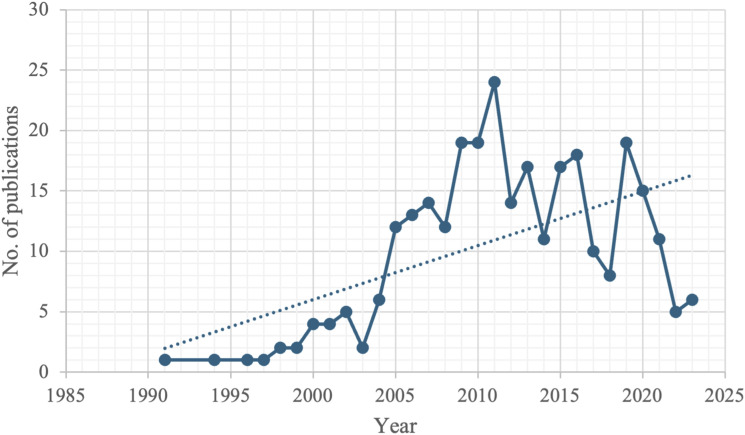
Annual trend of published articles.

The trend in the annual number of publications on MiECC is shown in Figure 3. A linearly increasing trend is visible from 1991 – the year of the first publication in this field.

Regarding journal metrics, the top 10 most productive journals are summarized in Table 1: *Perfusion* (2022 impact factor: 1.2) was the most represented journal, with a total of 71/232 (30.6%) of the published documents.

**Table 1.** Top 10 most productive journals and metrics.

Journal	Impact factor^a^	Best quartile^a^	Articles (*n*)
Perfusion-UK	1.2	Q4	71
Annals of thoracic surgery	4.6	Q1	26
European journal of cardio-thoracic surgery	3.4	Q1	26
Artificial organs	2.4	Q3	26
ASAIO journal	4.2	Q2	20
Interactive cardiovascular and thoracic surgery	1.7	Q3	14
Journal of thoracic and cardiovascular surgery	6.0	Q1	10
Journal of thoracic disease	2.5	Q3	8
Journal of cardiothoracic surgery	1.6	Q3	8
Thoracic and cardiovascular surgeon	1.5	Q3	7
Heart surgery forum	0.6	Q4	7

Legend.

^a^Source: Journal Citation Reports^TM^ 2022.

In terms of topics dealt with for each of the retrieved articles, the prevalence of various topics is shown as reported in the WOS database in Supplemental Figure 1 (Supplementary Material, Supplemental Figure 1). Most articles focused on transfusion requirements (30%), acute kidney injury (16%), inflammatory markers and cytokines (13%), inflammation (10%) and delirium (10%).

### Analysis of author cooperation network

An analysis of the author cooperation network was conducted to evaluate collaborative relationships among authors within the domain of MiECC. This involved identifying patterns of co-authorship, quantifying the extent of collaboration and co-citation, and analyzing the structure and dynamics of the network formed by authors and their collaborative ties. The aim was to elucidate knowledge dissemination and interdisciplinary collaboration within this field.

#### Bibliometric analysis of the keywords

**Figure 4.**
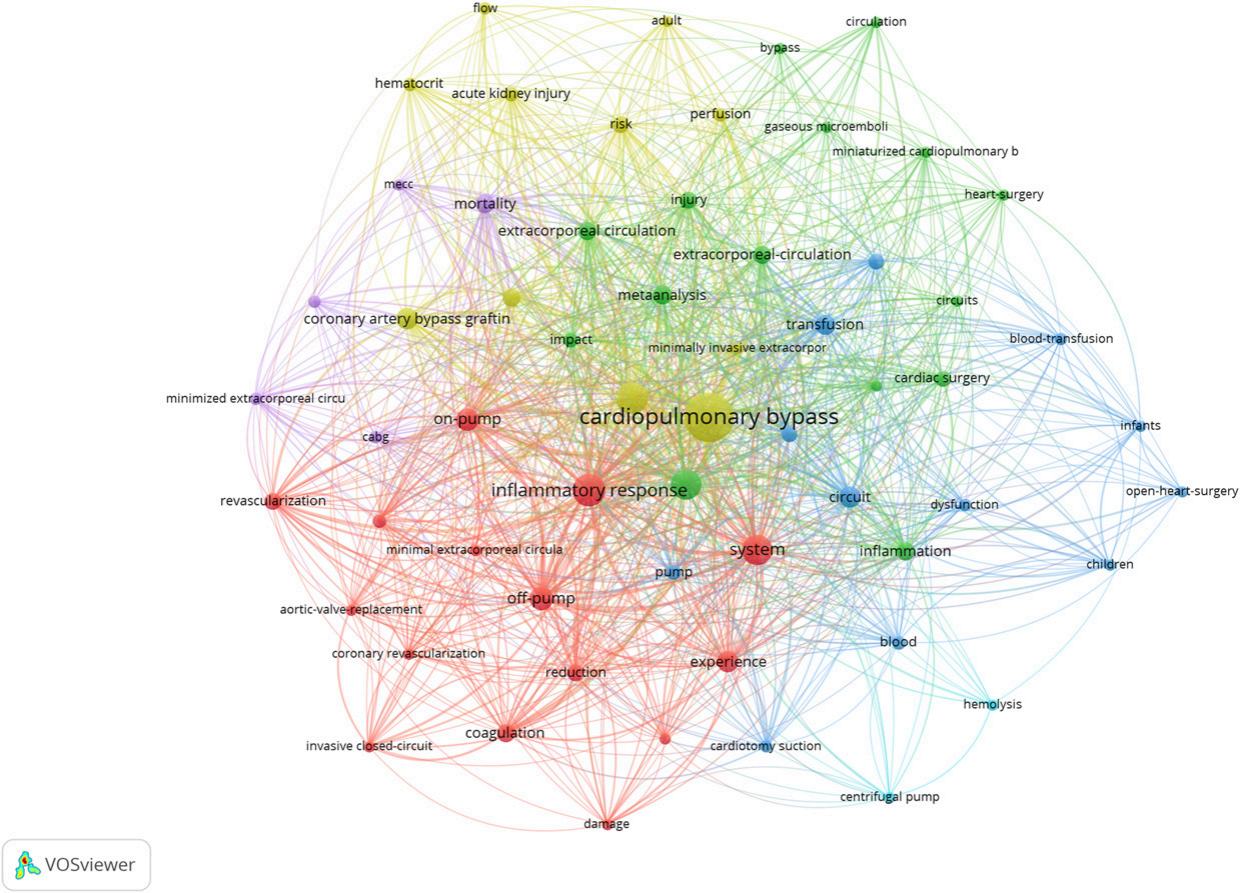
Network visualization map for cluster analysis based on co-occurrence of keyword analysis on minimal invasive extracorporeal circulation (MiECC). The size of the circles (nodes) indicates the frequency of occurrence. The curves between the nodes represent their co-occurrence in the same publication. The shorter the distance between two nodes, the larger the number of co-occurrence of the two keywords. The analysis provided 973 keywords, of which 60 met the threshold (8 items) and 6 clusters were obtained (different colors).

The analysis concerned keywords provided by authors which occurred more than 8 times in the WOS core database. Out of 973 keywords, 60 met the threshold (8 items) and 6 clusters, also referred to as ‘community’ in the literature and defined as a set of nodes which are closely related, were obtained. The keywords that appeared most were ‘CARDIOPULMONARY BYPASS’ (occurrence: 155; total link strength: 634), ‘INFLAMMATORY RESPONSE’ (68/330) and ‘CARDIAC SURGERY’ (63/282) (Figure 4).

#### Co-citation analysis for sources (journals)

A descriptive analysis of the top 10 co-cited articles^15–24^ is reported in Table 2. For the co-citation study, we used cited sources (i.e., journals) as a unit of analysis. The minimum number of citations from a source was 20. Of the 84 sources, 46 met the threshold. For each of these 46 sources, the total strength of the co-citation links with other sources was also calculated. *The Annals of Thoracic Surgery* obtained 1207 citations and 28,329 links; *Journal of Thoracic and Cardiovascular Surgery* had 719 citations and 21,138 links; *European Journal of Cardio-Thoracic Surgery* exhibited 577 citations and 13,698 links (Supplementary Material, Supplemental Figure 2).

**Table 2.** Top 10 cited articles.

First author ^Ref^	Title	Journal	Year	Topic	Citations
Wiesenack, C^18^	Four years’ experience with a miniaturized extracorporeal circulation system and its influence on clinical outcome	Artificial organs	2004	Engineering, biomedical; transplantation	135
Remadi, JP^15^	Prospective randomized study comparing coronary artery bypass grafting with the new mini-extracorporeal circulation jostra system or with a standard cardiopulmonary bypass	American heart journal	2006	Cardiac & cardiovascular systems	120
Bronicki, RA^17^	Cardiopulmonary bypass-induced inflammatory response: pathophysiology and treatment	Pediatric critical care medicine	2016	Critical care medicine; pediatrics	115
Anastasiadis, K^22^	Use of minimal invasive extracorporeal circulation in cardiac surgery: principles, definitions and potential benefits. A position paper from the minimal invasive extra-corporeal technologies international society (MiECTiS)	Interactive cardiovascular and thoracic surgery	2016	Cardiac & cardiovascular systems; respiratory system; surgery	113
Biancari, F^16^	Meta-analysis of randomised trials comparing the effectiveness of miniaturised versus conventional cardiopulmonary bypass in adult cardiac surgery	Heart	2009	Cardiac & cardiovascular systems	84
Anastasiadis, K^19^	Neurocognitive outcome after coronary artery bypass surgery using minimal versus conventional extracorporeal circulation: a Randomised controlled pilot study	Heart	2011	Cardiac & cardiovascular systems	76
Wippermann, J^20^	Comparison of minimally invasive closed circuit extracorporeal circulation with conventional cardiopulmonary bypass and with off-pump technique in CABG patients: selected parameters of coagulation and inflammatory system	European journal of cardio-thoracic surgery	2005	Cardiac & cardiovascular systems; respiratory system; surgery	76
Nollert, G^21^	Miniaturized cardiopulmonary bypass in coronary artery bypass surgery: Marginal impact on inflammation and coagulation but loss of safety margins - invited commentary	Annals of thoracic surgery	2005	Cardiac & cardiovascular systems; respiratory system; surgery	70
Zangrillo, A^23^	Miniaturized cardiopulmonary bypass improves short-term outcome in cardiac surgery: A meta-analysis of randomized controlled studies	Journal of thoracic and cardiovascular surgery	2010	Cardiac & cardiovascular systems; respiratory system; surgery	67
Huybregts, RAJM^24^	Attenuated renal and intestinal injury after use of a mini-cardiopulmonary bypass system	Annals of thoracic surgery	2007	Cardiac & cardiovascular systems; respiratory system; surgery	67

#### Co-authorship analysis (countries)

The co-authorship analysis was conducted by considering 5 as the cut-off number for the minimum number of documents having the first author from a specific country. Consequently, from a total of 36 countries, 16 met the threshold. In Germany our analysis recorded 97 published documents and 41 links; in Italy, 29 documents and 26 links; in England, 29 documents and 25 links (Supplementary Material, Supplemental Figure 3).

#### The most productive organizations and their collaborations

The minimum number of documents (threshold) for affiliations (i.e., organizations) was 5. Of 359 organizations, 18 met the threshold. For each of these 18 organizations, the total strength of the citation links with other organizations was calculated. The ‘UNIVERSITY OF BERN’ (Switzerland) produced 10 documents and a total link strength of 2375; the ‘AHEPA UNIVERSITY HOSPITAL’ (Greece) published 30 documents and 2249 links; the ‘OULU UNIVERSITY HOSPITAL’ (Finland) published seven documents and 2104 links; the ‘UNIVERSITY OF REGENSBURG’ (Germany) 15 documents and 2066 links; the ‘KLINIKUM BRAUNSCHWEIG’ (Germany) published 10 documents and 1537 links; the ‘UNIVERSITY OF ULM’ (Germany) published 5 documents and 1252 links (Supplementary Material, Supplemental Figure 5).

### Co-citation analysis (authors)

**Figure 5.**
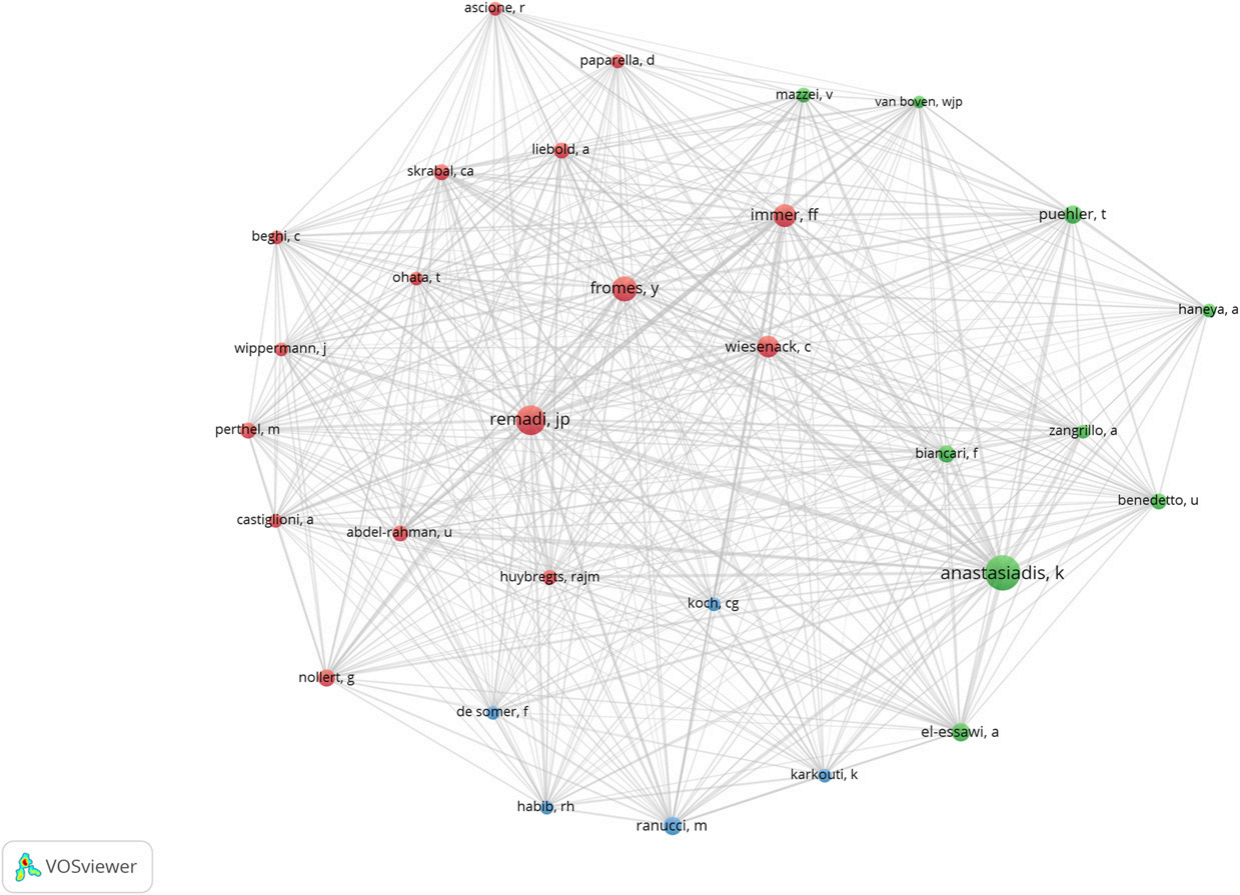
Co-citation analysis for authors. The size of the circles (nodes) indicates the number of citations for each author. The lines between the nodes represent their co-citation links with other authors. A co-citation link is a link between two items (authors) that are both cited by the same document. The analysis provided 3286 authors, of which 30 authors met the threshold (25 citations) and 3 clusters were obtained (different colors), each encompassing authors with close cooperative relationship.

For the co-citation study, we considered the author as a unit of analysis. In this case, the minimum number of an author’s citations was 25. Out of 3286 authors, 30 met the threshold, and for each of them, the total strength of the co-citation links with other authors was calculated. Anastasiadis K was the most cited author with 189 citations and 1331 total link strength, followed by Remadi JP, who obtained 134 citations and 1172 links. Thirdly, Fromes J had 94 citations and 740 links. Fourthly, Immer FF had 77 citations and 724 links. Last, Wiesenack C had 71 citations and 576 links (Figure 5).

## Discussion

Although systematic reviews and meta-analyses of MiECC have been previously performed and are available in the published literature, as far as we know, this is the first bibliometric network analysis that specifically focuses on this subject. Notably, bibliometric studies stand as an ideal research methodology for analyzing past and current trends on critical medical topics and provide researchers with additional ideas about new studies they can design.^9^

In recent years, trend analysis has revealed a continuous rise in the scientific community’s interest in MiECC. Since 1991, when the first scientific publication on MiECC was released, a consistent annual increase in the number of published documents has occurred. This growth may indirectly reflect the significant research advancements made in this field over recent decades. However, the widespread adoption of MiECC rather than CPB in routine clinical practice remains limited. This may be primarily due to the ongoing debate surrounding the former’s precise clinical advantages and the absence of a definitive consensus on its safe and appropriate use in various cardiac surgery procedures. One of the primary criticisms of MiECC is that many surgeons view it primarily as an alternative to off-pump surgery, thereby restricting its application to coronary artery bypass grafting (CABG) procedures. Only a minority of MiECC programs have so far extended its use to aortic valve surgery, with only a handful of teams worldwide advocating for its universal applicability across all cardiac surgeries. As such, incorporating a ‘safety net’ into MiECC (Type IV) may further facilitate its implementation in complex cardiac surgery as well. Therefore, further studies are warranted to ascertain whether MiECC can be universally employed across diverse forms of cardiac surgery.

Notably, approximately one-third of the publications revolve around the primary emphasis on MiECC’s ability to mitigate allogenic blood product transfusions (Supplementary Material, Supplemental Figure 1). MiECC exhibits reduced hemodilution and, particularly with its Type II and III designs, minimizes contact between blood and non-endothelial surfaces as well as air. Moreover, when combined with the separation of pleuro-pericardial aspirations, it shows potential to require a lower degree of systemic heparinization. Notwithstanding, this aspect may appear to be still insufficiently explored and addressed in published studies and constitutes a promising avenue for further investigation and understanding within the field of MiECC research.

Furthermore, it appears that the economic viability of MiECC is a relatively overlooked area in the existing literature. We contend that conducting more cost-utility analysis is essential to evaluate the financial sustainability of incorporating MiECC into routine clinical practice.

Nevertheless, the majority of the articles are published in high-ranked journals, indicating that strengths, limitations and potential viability of MiECC in daily clinical practice represent a central topic in the field of ECC research. Of note, some journals have shown a keen interest in addressing these issues. For instance, *Perfusion*, *The Annals of Thoracic Surgery*, and *European Journal Cardio-Thoracic Surgery* have published 71, 26 and 26 papers, respectively. More specifically, *Perfusion* contributed the highest number of published documents, accounting for more than one-third of the total number of publications. This observation may indirectly reflect that researchers have long perceived *Perfusion* as the leading target journal for their investigations in the field of MiECC. Consequently, it’s advisable for authors intending to publish on MiECC to primarily focus on these journals. Additionally, *The Annals of Thoracic Surgery* emerged as the most-cited journal, with 1207 citations and 28,329 links.

Furthermore, since a high number of citations reflects the quality of the articles, we suggest that clinicians and researchers interested in the topic of MiECC carefully scrutinize the top 10 cited publications first.^15–24^

Semantic network exploration serves as a pivotal element in network analyses, as it clarifies the research objectives within a specific field and unveils potential new findings. Remarkably, the network visualization of the most frequently cited keywords (Figure 4 which describes the connections between terms reveals potential research gaps in several crucial areas. Specifically, a lack of studies exploring the optimal anesthetic management during MiECC and focusing on administered drugs and their role in preventing common complications (i.e., vasoplegia and low-cardiac output syndrome (LCOS)) arose. On this front, while the incidence of complications (e.g., bleeding, arrhythmias, pulmonary insufficiency resulting from fluid overload, renal dysfunction, etc.) may be reduced with MiECC compared to conventional ECC techniques, such advantage cannot be directly extrapolated by the present bibliometric network analysis. Furthermore, given that the basic anesthetic management of both conventional and minimal invasive ECC perfusion techniques is very similar, other critical topics, including the intraoperative necessity for inotropes, vasopressors, and fluids to maintain adequate blood flow and perfusion pressure during MiECC, show potential to differ between these two perfusion techniques and were also found to be under-explored. Provided that these factors may all, albeit indirectly, impact morbidity, mortality, and healthcare costs, further investigation in the future is warranted.

The research results are distributed across various countries and the majority of the co-authorship network involves Germany, Greece, Italy, and England. Upon examination of collaborations between research centers, a significant deficiency emerged – the absence of an international, multi-centric, two-group, parallel RCT focusing on the use of MiECC in the most common types of heart surgery. Four meta-analyses which comprise some overlapping RCTs have shown substantial advantages in the use of MiECC, particularly in terms of reduced 30-day mortality and the incidence of stroke, myocardial infarction, post-operative atrial fibrillation, and renal dysfunction.^16,25,26^ Notably, no adverse effects were reported. However, akin to conventional ECC perfusion systems, variations in MiECC circuit designs, patient characteristics, and study endpoints, along with less-than-ideal methodological quality in many of the included RCTs, have the potential to diminish the strength of such evidence.

As such, it is the authors’ opinion that for a new perfusion strategy to become widely accepted, it needs to be easily replicated. Our analysis of bibliometric data reveals that the majority of current body of research on MiECC primarily originates from a small set of research groups only, often referring to and citing each other’s work. While the concentration of research groups in a select few countries doesn’t necessarily undermine scientific data quality, it may indirectly suggest that performing MiECC effectively might demand a high level of expertise. Therefore, to broaden the acceptance of MiEEC, it’s essential to engage a more diverse range of researchers and demonstrate its significant advantages over conventional ECC perfusion methods. In the absence of these efforts, many hospitals may persist in utilizing traditional CPB for on-pump cardiac surgery.

Nevertheless, the potential advantages of MiECC underscore the pressing need for a comprehensive, high-quality RCT to assess this technology. In this regard, a Europe-wide, multi-center RCT – the COMICS trial – was conducted. The trial aimed to assess the effectiveness of MiECC versus conventional CPB in 3500 patients undergoing CABG, aortic valve replacement (AVR), or CABG + AVR surgeries. Although initiated in May 2018 and completed in 2021, the results of the trial have not yet been published.^26^ As such, given that – to date – the most impactful studies do not originate from multi-centric collaborations, it is crucial to establish additional networks for further research in this field.^27^

### Study limitations

This is the first bibliometric analysis offering insights drawn from current global evidence on the emerging subject of MiECC. Nonetheless, it is important for readers to bear in mind certain study limitations, as bibliometric studies cannot replace systematic reviews or meta-analyses.

To begin with, a bibliometric analysis does not yield conclusive findings for evidence-based medicine. Nevertheless, when applied to bibliometric datasets, clustering techniques provide a visually effective overview.

Additionally, a notable limitation of this analysis concerns the time factor: newer publications have had fewer opportunities for citations in subsequent works compared to earlier-published papers. Therefore, while the current findings are subject to change, our contribution presents an appealing ‘snapshot’ of previously published data at the time of the present publication.

Furthermore, another constraint of this analysis pertains to the structure of the underlying network. The connections between publications rely on citations or word associations. As a result, keywords, the frequency of words cited throughout the text, affiliations, and other factors exert a substantial influence on the representation of the outcomes.

## Conclusion and future perspectives

The present bibliometric network analysis of MiECC can serve as a valuable tool for shaping future research endeavors in this field. While our investigation has shed light on the most prominent scholars, trending topics and core research teams, it has also revealed several gaps in current knowledge that warrant attention.

For instance, in upcoming research, it would be beneficial to focus on exploring the influence of MiECC on hard clinical outcomes. Although the majority of studies in the published literature have addressed separately certain clinical outcomes (e.g., transfusion requirements) the absence of a comprehensive and holistic examination of MiECC’s effects on concrete, tangible clinical outcomes may have somewhat impeded the integration of this perfusion technique into routine clinical practice.

Additionally, the practical experience with MiECC appears to be limited to few selected centers worldwide. Consequently, in order to promote broader adoption of this novel perfusion technique, it is imperative to establish additional networks and conduct high-quality clinical studies.

## Supplemental Material


Supplemental Material - Minimal invasive extracorporeal circulation: A bibliometric network analysis of the global scientific output
Supplemental Material for Minimal invasive extracorporeal circulation: A bibliometric network analysis of the global scientific output by Jacopo D’Andria Ursoleo, Rosario Losiggio, Margherita Licheri, Gaia Barucco, Stefano Lazzari, Carolina Faustini and Fabrizio Monaco; Collaborators in Perfusion.

## Data Availability

Further information is available from the corresponding authors upon reasonable request.*
