# Supplementary material for: Minimal invasive extracorporeal circulation: A bibliometric network analysis of the global scientific output
Source: Perfusion. 2024 Sep 17;40(5):1176–86. doi: 10.1177/02676591241269729 (PMC12202833; doi:10.1177/02676591241269729)

SUPPLEMENTARY MATERIAL TO:

Minimal Invasive Extracorporeal Circulation: A Bibliometric Network Analysis of the Global Scientific Output

CONTENTS

Supplemental Figure 1 p. 2

Supplemental Figure 2 p. 3

Supplemental Figure 3 p. 4

Supplemental Figure 4 p. 5

Supplemental Figure 1 The most frequently used topics in articles on minimal invasive extracorporeal circulation based as per the Web of Science. One or more topics were recorded for each article.

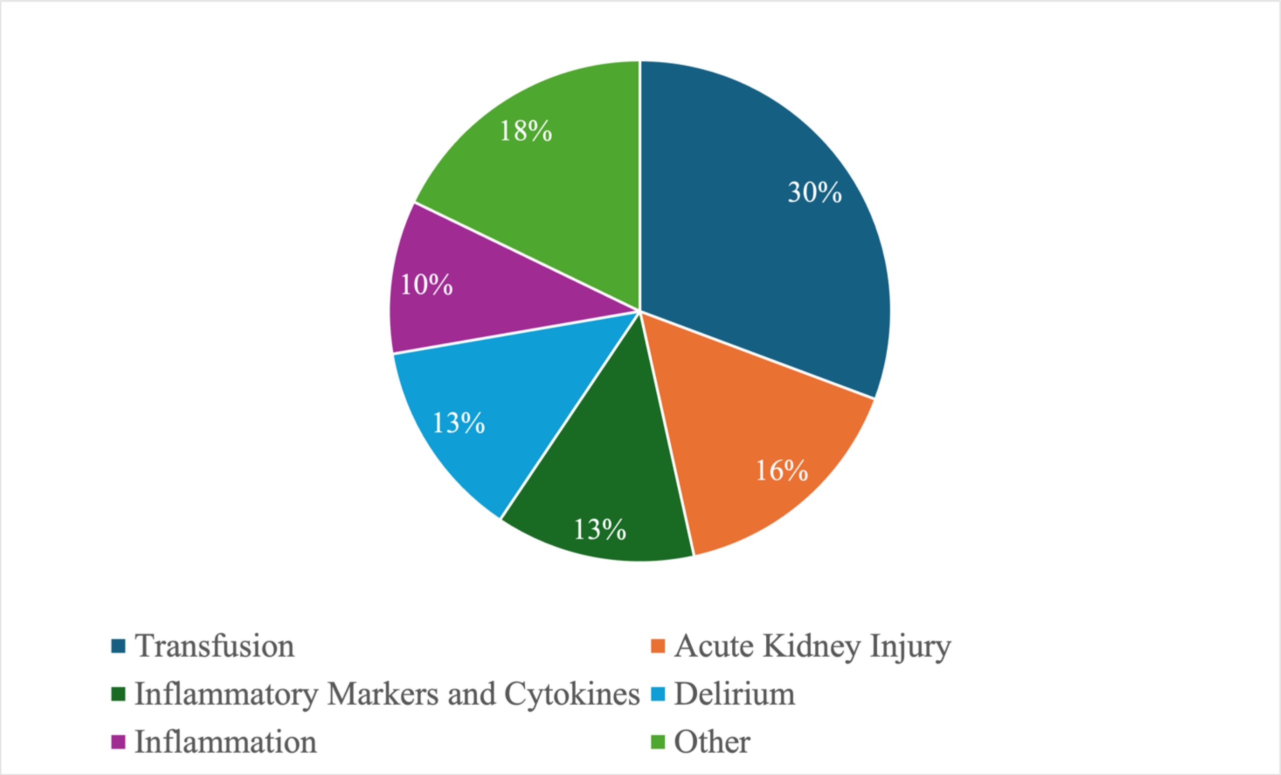

Supplemental Figure 2 Co-citation analysis visualization map for sources (i.e., journals). The size of the circle indicates the large number of articles.

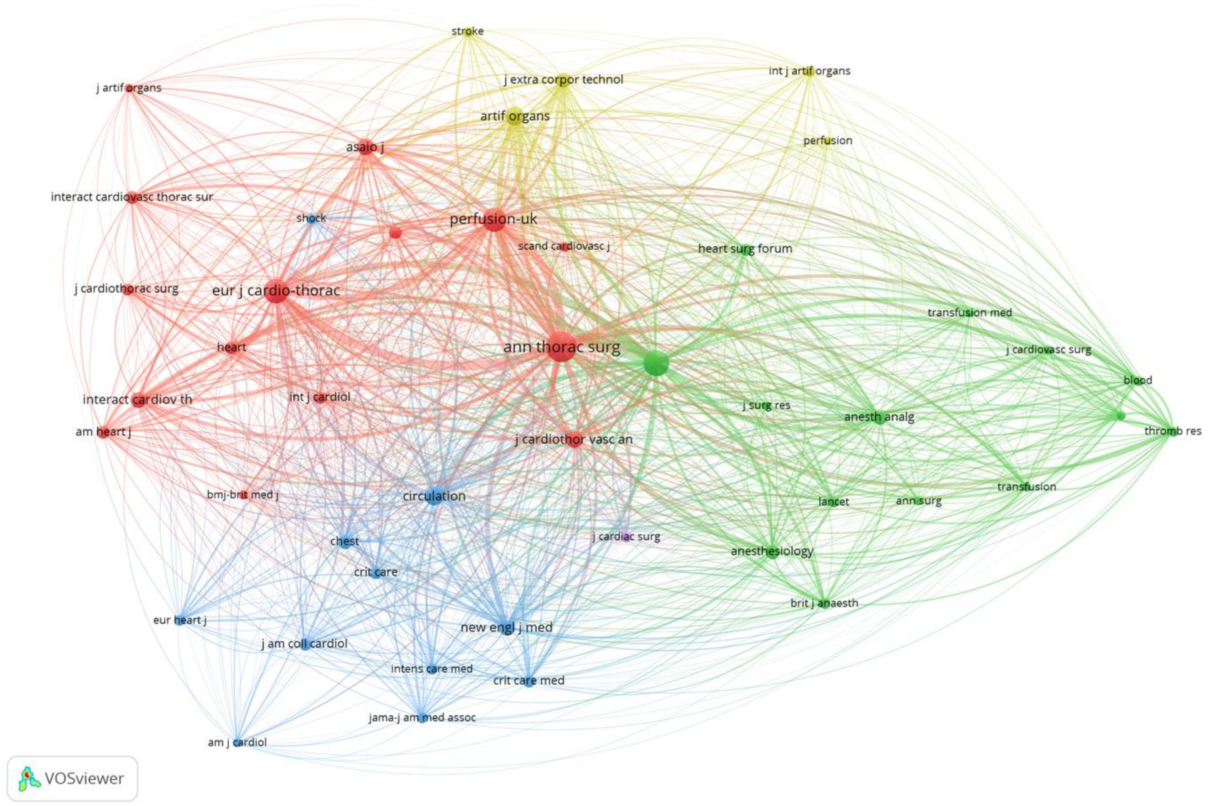

Supplemental Figure 3 Co-authorship analysis for countries. Four countries reached the threshold of ~~10~~ 5 documents. Clusters are organized based on year intervals.

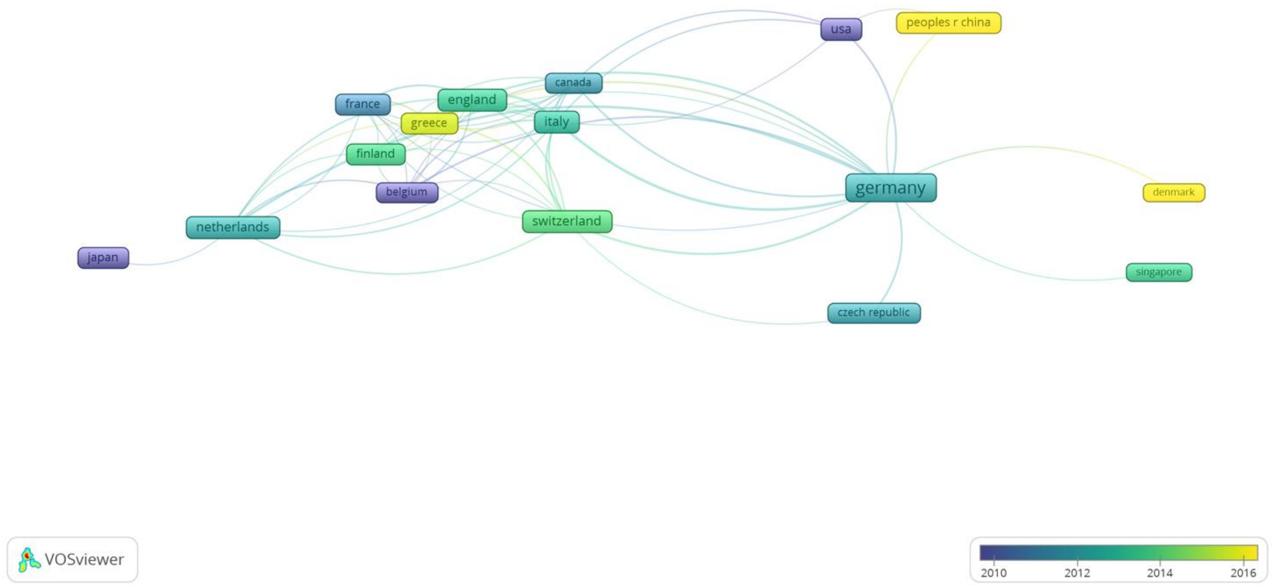
usa
peoples r china
canada
france
england
greece
italy
finland
germany
belgium
denmark
netherlands
switzerland
japan
singapore
czech republic
VOSviewer
2010
2012
2014
2016

Supplemental Figure 4 The most productive organizations and collaboration links between institutions on minimal invasive extracorporeal circulation (MiECC). The size of the circle indicates the large number of articles.

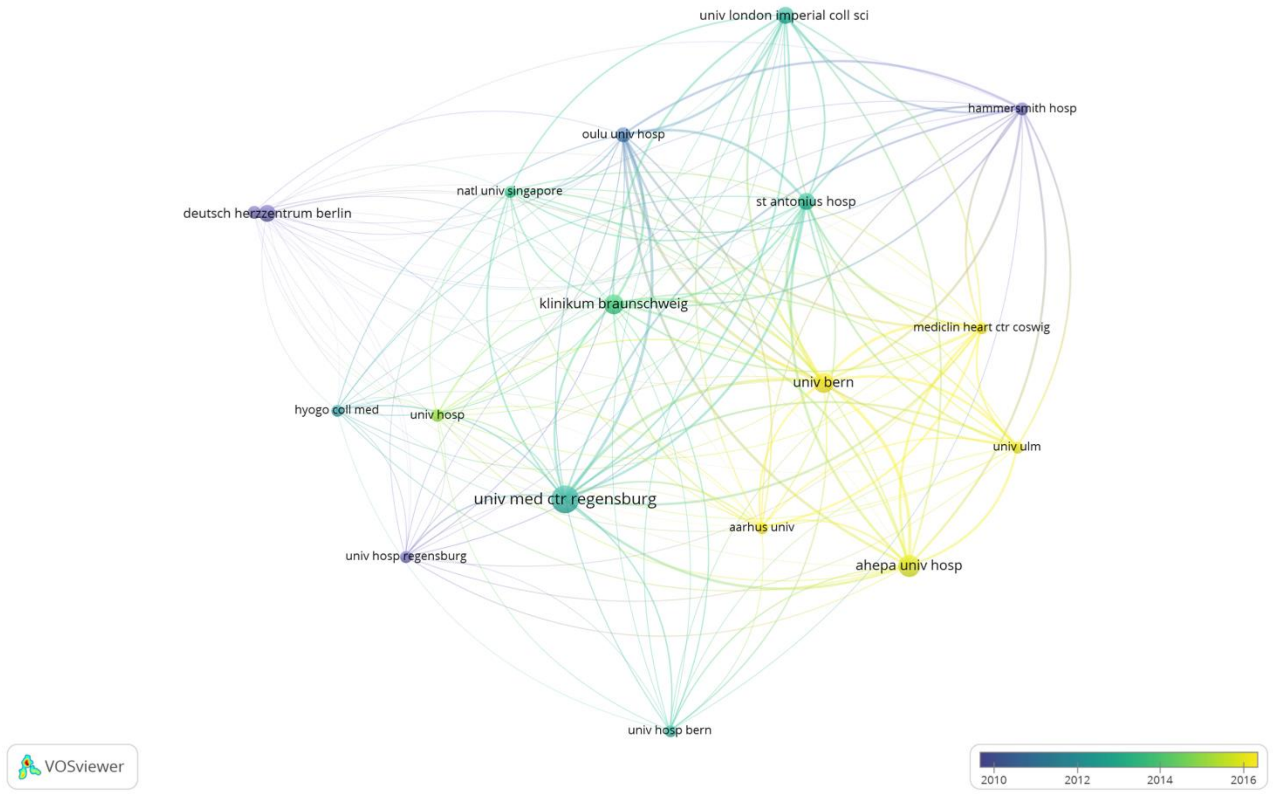

Supplement: Supplemental Material - Minimal invasive extracorporeal circulation: A bibliometric network analysis of the global scientific output [file sj-pdf-1-prf-10.1177_02676591241269729.pdf]
